# Exodus of Lebanese doctors in times of crisis: a qualitative study

**DOI:** 10.3389/frhs.2023.1240052

**Published:** 2023-10-30

**Authors:** Elie Nemr, Marianne Moussallem, Rita Nemr, Michèle Kosremelli Asmar

**Affiliations:** ^1^ Faculty of Medicine, Saint Joseph University, Beirut, Lebanon; ^2^ Higher Institute of Public Health, Faculty of Medicine, Saint Joseph University of Beirut, Lebanon; ^3^ Department of Endocrinology, Lebanese American University, Beirut, Lebanon

**Keywords:** doctors, migration, retention, push and pull factors, health workforce

## Abstract

**Introduction:**

Since 2019, Lebanon is experiencing an unprecedented exodus of doctors, seriously threatening the national health system, which is expected to continue without quick and effective solutions. Therefore, this study aimed to understand the factors that push Lebanese doctors to migrate and the factors that retain others in the country. Additionally, this study aims to propose solutions to preserve an adequate supply of medical care amidst the crisis.

**Methods:**

Qualitative semi-structured interviews and focus group discussions were conducted using pre-developed guides. Purposive and snowball sampling was adopted to recruit physicians who emigrated and physicians staying in Lebanon. Transcripts of interviews and focus groups were coded using Dedoose software and analyzed through a combination of inductive and deductive approaches.

**Results:**

Emigration was found to be the result of numerous interconnected factors. The main drivers for emigration were declining income, career problems, reduced quality of care, unhealthy work environment, and the deteriorated political and socio-economic contexts leading to instability and insecurity. As for the retention factors, they included affective attachment and sense of belonging to the professional environment and the country, followed by recognition and valorization at work. Several recommendations were developed to maintain quality of care delivery, including reforms of the health system, development of focused human resource retention strategies based on resource mapping evidence, negotiations with recruiting institutions to endorse the code ethics ending unethical practices draining countries' human resources, provision of financial incentives to doctors, and the recognition and valorization of physicians. Other rapid interventions were suggested, such as short-term medical missions to mitigate shortages in certain specialties, telemedicine, adaptation of recruitment processes to compensate for resources shortages in certain specialties, and adoption of task-shifting approaches to alleviate the workload on overburdened specialists.

**Discussion:**

The findings of this study shed the light on the different factors influencing migration while framing them in the Lebanese context. These findings and recommendations should inform stakeholders and policy makers about the interventions needed to restore the quality of care. The feasibility and sustainability of most formulated recommendations depend on several factors, with political and socio-economic security and stability being the most crucial ones.

## 1. Introduction

The migration of physicians profoundly affects health care delivery by creating imbalances in terms of numbers, available expertise, and deployment of human resources at country level, which results in fragile and underperforming health systems (1, 2). Also known as doctors' “brain drain” (3), the pattern of this phenomenon is well known, following an influx of health workforce from low-resource countries to more developed ones (4).

The emigration of physicians is a multifaceted phenomenon shaped by a complex interplay of factors. These factors encompass a wide spectrum, ranging from national considerations, including a nation's political and socio-economic context, to professional aspects within the healthcare system. Additionally, individual physicians' career aspirations, the challenges they face in pursuing professional growth, and personal and familial considerations all play a significant role in driving this phenomenon.

Various models within the literature have attempted to categorize these factors into comprehensive frameworks. For instance, Dohlman et al. employed Maslow's hierarchy of needs, dividing these factors based on the needs they address, such as physiological, safety, social, esteem, and self-actualization needs. These levels were seen as interdependent, with higher-level needs building upon the fulfillment of lower-level ones (4). Furthermore, a systematic review conducted by Blacklock et al. classified factors into “push” factors, which propel physicians to emigrate, and “pull” factors which attract physicians to specific destinations (5). A third interesting component, which was also described as affecting physicians' migration by retaining them in their country of origin, is the “stick and stay” factors (6). Evidence suggests that “Low wages, poor job satisfaction and motivation, persistent shortage of basic supplies, dangerous working conditions, outdated equipment, lack of supervision and postgraduate training, limited career opportunities, and lack of employment opportunities” (2) are the main drivers *push factors*. As for the factors retaining physicians in the country of origin—*stick and stay factors*—they mainly encompass comfort of children in current country and the social belonging (6). Nonetheless, although physicians were pushed to emigrate rather than incited to immigrate (7), a range of *pull factors* were described in the literature and include: the quality of the health system in terms of organization and infrastructure, physicians' remuneration, education and career opportunities, better quality of life and living standards, socio-economic and political stability, etc. (3, 7). Several scholars have approached the topic of physician migration using the macro-, meso-, and micro-levels of analysis (8, 9). For instance, Hajian et al. classified these factors into three overarching domains in their study, with macro-level factors encompassing a nation's economic, political, and social determinants. These factors include income disparities, political instability, and societal inequalities, such as gender and ethnic discrimination, all of which play a pivotal role in influencing migration decisions. The study also delves into the meso-level, meticulously investigating the educational and professional environment that physicians navigate. This involves a comprehensive analysis of post-graduate training opportunities, including their availability and competitiveness, as well as the clarity and quality of educational curricula. The study also examines professional opportunities, scrutinizing career prospects, job market dynamics, and access to cutting-edge technologies and resources, all of which significantly influence the decision to migrate. Finally, the investigation describes the micro-level factors, where deeply personal and familial motives underpin migration decisions. These factors encompass a wide range of considerations, from the pursuit of adventure and improved quality of life to financial aspirations, language acquisition, and responses to partner or parental decisions.

The migration of doctors means that the resources invested by low-resource countries to educate and train these young doctors have been wasted without benefiting the country (2, 3, 10, 11). In addition, the resulting collapse of the health care system will hamper further development and economic growth. Thus, states are invited to promptly curb the “brain drain” phenomenon and mitigate its consequences, by adopting focused retention strategies. Possible retention strategies described in the literature included improving trainings and increasing employment opportunities through strengthened involvement and increased support from hospitals and universities, improvement of working conditions in country of origin, facilitated return (2), but most importantly financial, social and political stability (4). This issue has also been addressed from a global perspective, the most recent intervention being the code of practice developed by the world Health Organization (WHO) in 2008. It consists of a “soft low” approach aiming at putting together high and low resource countries with the purpose of regulating doctors' migration and preventing unethical practices mainly on the side of destination countries (2, 12, 13). However, until 2017, only a few Member States had changed their policies to comply with the recommendations of the code of practice (2).

The migration of doctors is a prevalent phenomenon in Lebanon. In fact, Lebanon already had the highest emigration rate among medical graduates working abroad in the Middle East and North Africa, and it ranked as the seventh highest in the world in terms of the approximate percentage of medical graduates practicing overseas (14). For instance, in 2004, approximately 40% of Lebanese past medical school graduates were practicing in the United States (15, 16). Furthermore, when considering the size of Lebanon's population, the country ranked second among nations with international physicians practicing in the United States who are graduates of Lebanese medical schools. This indicates the already high density of migration from Lebanon to more resourced countries. Nevertheless, in 2021, the President of the Lebanese Order of Physicians warned that Lebanon is witnessing an unprecedented exodus of physicians, with more than 2,500 doctors most of them being aged between 35 and 55 having left the country to settle abroad. This remarkable emigration that seriously threatens the local health system and medical training is believed to be the result of the unparalleled socio-economic crisis that the country has been grappling since October 2019. This crisis has been so severe that it led to Lebanon's reclassification as a lower-middle-income country by the World Bank, marking a significant decline from its previous upper middle-income status, a change that occurred in July 2022 (17). The World Bank has also recognized this crisis as one of the top ten worst episodes globally since the mid-19th century. This situation was further aggravated by the COVID-19 pandemic and the explosion in the port of Beirut on August 4th, 2020. The cumulative impact of all these crises has deeply affected the “inputs” of the health system, jeopardizing the delivery of services at the national level. In fact, Lebanon, previously recognized as a “prominent tertiary medical hub in the Middle East” and a secondary care-focused healthcare system (18), has witnessed the deterioration of the main pillars supporting this system: infrastructure and human resources (19). For instance, during his visit to Beirut in 2021, the Director General of the World Health Organization (WHO), expressed his “deep concern” about the impact of the socio-economic crisis on the health of the Lebanese and announced the commitment of the international organization to cooperate with its partners to re-strengthen the system. However, whatever support is received, it is crucial to understand the roots of the causes of the problem and to deal with them. In fact, evidence suggests that the severe devaluation of the national currency led to a sharp decline in the value of wages, which, together with the lack of incentives to retain human resources, resulted in a mass emigration of competent health workers (19). Therefore, this study aimed to understand through a holistic approach the factors that push Lebanese doctors to migrate or to consider migrating, the factors that push certain others to stay in the country or to return (for migrants) as well as to propose measures to preserve a supply of adequate medical care amidst the crisis.

## 2. Methodology

The researchers used qualitative methods consisting of semi-structured interviews and focus group discussions (FGD). The semi-structured interviews were conducted with physicians who had left the country or were in the process of emigrating, as well as those who had chosen to stay in the country. These interviews aimed to understand the factors influencing their decisions. As for the FGD, they involved another group of physicians representing both categories. The FGDs were designed as co-creation exercises, intended to facilitate discussions based on the findings from the qualitative interviews. Their primary goal was to develop a set of recommendations aimed at maintaining the quality of care provision amidst the Lebanese crisis. The semi-structured interviews and focus group discussions were conducted from July to September 2022.

### 2.1. Semi-structured interviews

#### 2.1.1. Data collection process

An invitation email was sent by the principal investigator (EN)—including an information sheet—to invite the key informants (KI) to participate in the study. The information sheet provided comprehensive details about the study's objectives, participation requirements, and the methods employed to ensure the anonymity of collected information and the confidentiality of informants. It also explicitly outlined the informants' right to withdraw from the study at any point. These points were reiterated before commencing the interviews to prevent any undue pressure on the informants, especially considering their familiarity with the principal investigator. Interviews were then conducted virtually using Microsoft Teams software with physicians who consented to participate. The interviews were administered by a single researcher (EN) in French and English (according to the preference of the informants) and were recorded after obtaining the consent of the interviewee. The interviews had an approximate duration of 45 min. The recordings were stored on Microsoft Teams in a folder accessible exclusively to the principal investigator (PI) and another researcher, MM. Following transcription, they were transferred to the Dedoose software, with each transcript labeled using the acronyms “MD” for doctors intending to emigrate or who had emigrated and “SD” for doctors opting to stay, followed by consecutive numbers.

#### 2.1.2. Key informants sampling

The principal investigator initially identified two groups from the university hospital in which they practice: physicians in the process of emigrating or those having already migrated and those choosing to stay in the country. It's important to clarify that “Physicians who decided to migrate” or “are in the process of emigration”, as selected for this study, are individuals who were leaving the country within a matter of weeks. In other words, these doctors had already made all the necessary arrangements and were in the process of departing. Following this, a purposive snowball sampling approach was utilized to expand the sample, with the goal of encompassing informants from various regions of practice, different specialties, and socio-demographic characteristics. This approach also helped recruit informants exercising in various settings outside the university hospital of the principal investigator. As for the inclusion criteria, they included (1) physicians consenting to participate and (2) physicians having left the country after October 2019 for the migrating doctors and doctors having decided to stay in the country for doctors staying in Lebanon. As for the exclusion criterion, it was related to the fact of having left the country before October 2019. Consequently, informants were requested to provide names and contact information of potential informants, who were subsequently contacted and invited to participate in the study by the principal investigator. A total of 28 interviews were carried out. The characteristics of informants are presented in Table 1.

**Table 1:** Key informants' characteristics.

Key informant category	Sample
Physicians who emigrated or decided to emigrate	Nineteen (19) semi-structured interviews were conducted with migrant doctors: – Eight (8) women and eleven (11) men. – Ten (10) were married among which eight (8) had children, seven (7) were single and two (2) were divorced. – The years of practice varied between one year and twenty-nine years (29).
Physicians who decided to stay	Nine (9) semi-structured interviews were conducted with doctors staying in Lebanon. – Four (4) women and five (5) men. – Seven (7) were married among which five (5) had children, two (2) were single. – The years of practice varied between five (5) year and twenty-five years (25).

#### 2.1.3. Data collection tools

The researchers created two distinct topic guides for the semi-structured interviews, one tailored for each category of physicians (those who have or plan to emigrate and those who have chosen to stay). These guides were developed based on two separate yet complementary frameworks: the theoretical framework developed by Hajian et al. (9) categorizing the factors influencing migration into three main levels (macro level, meso level, and micro level), as well as the “push and pull” and “stick and stay” factors approach explaining the “brain drain” of doctors described in a study conducted by Oberoi and Lin (6). The different guides covered the following items: (1) the socio-economic status of participating physicians, their professional experience, dual nationality status, (2) the factors influencing their decision (to emigrate or to stay in Lebanon), (3) factors that would push them to return (for those who migrated) or push them to emigrate (for those staying in Lebanon), and (4) suggestions for maintaining quality medical care amidst the current Lebanese crisis. The topic guides were developed in English and translated into French. They are available as Supplementary Material.

#### 2.1.4. Data analysis

**Figure 1:**
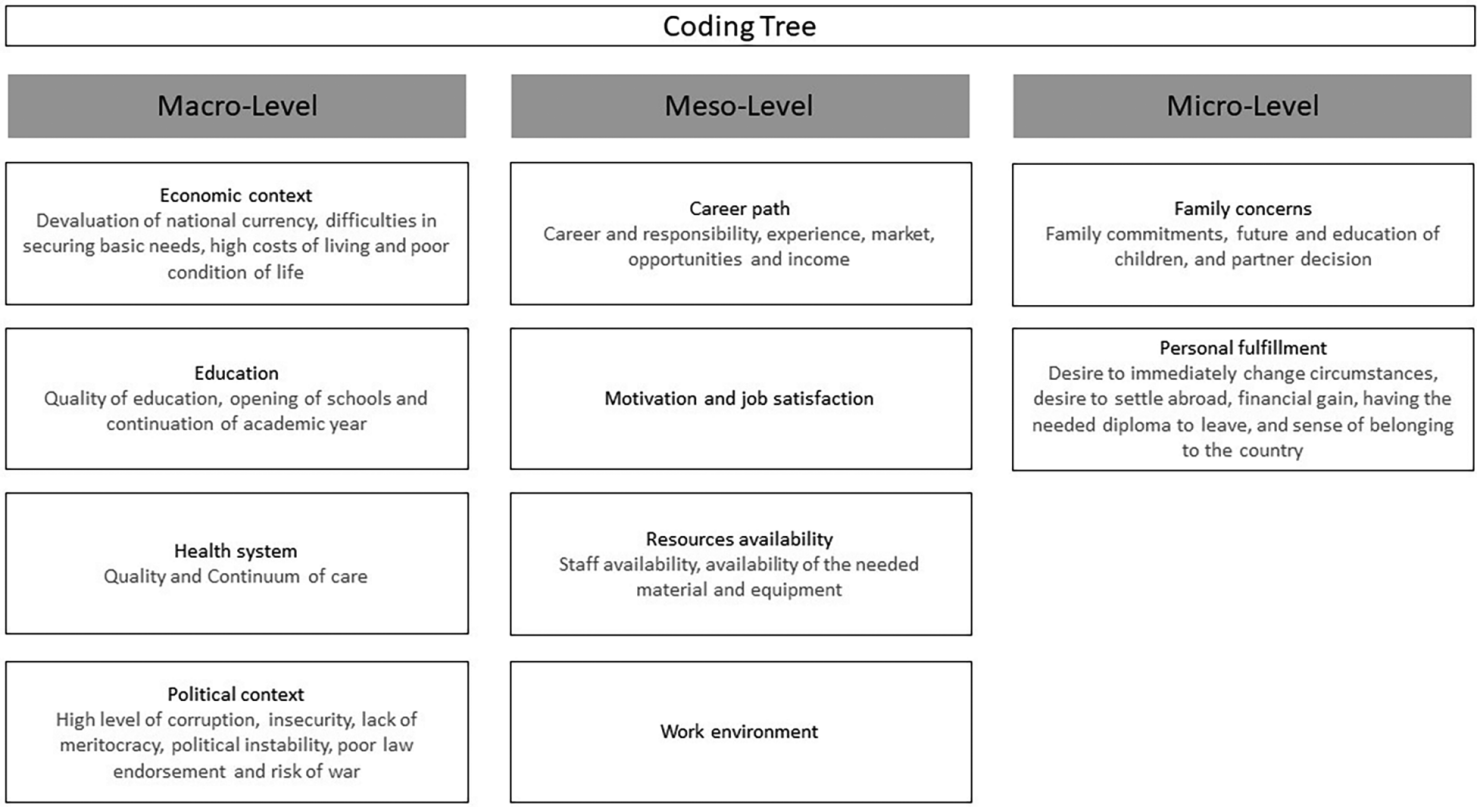
Final coding tree.

All interviews were transcribed in English and uploaded onto Dedoose software for coding and analysis. Both inductive and deductive data analysis approaches were employed. Initially, the researchers (EN, MM) generated a set of codes using Hajian et al. theoretical framework (9). The coding and the thematic analysis were conducted by EN and MM concurrently with the data collection process with regular discussion between the two researchers to mitigate any potential bias stemming from the principal investigator's positionality. As the coding process progressed, additional codes were created to capture emerging themes from the data. The finalized coding tree is illustrated in Figure 1. The analysis and finalization of interview findings were concluded before conducting the FGD.

### 2.2. Focus group discussions

#### 2.2.1. Data collection process

The researchers identified and included a new group of physicians consisting of both emigrating doctors and those who had made the decision to stay. Two discussion groups were organized: (1) the first FGD was conducted virtually and included physicians who migrated (2) the second FGD was conducted in person and gathered a group of doctors who had chosen to stay in Lebanon. The FGD were moderated by the principal investigator (EN), they were conducted in French and only the first FGD was recorded given that one of the doctors participating in the second FGD refused having the discussion recorded. During the unrecorded FGD, a designated notetaker was responsible for documenting the discussions and formulated recommendations. The moderator (EN) commenced each session with a debriefing of the semi-structured interview findings, inviting participants to offer practical recommendations to address the factors driving doctors to leave and to further leverage the elements retaining physicians in the country. Subsequently, EN concluded each session by summarizing the main recommendations provided. Each FGD had an approximate duration of one hour. Please consult the Supplementary Material for specific information regarding the proceedings and structure of the FGDs.

#### 2.2.2. Participants sampling

A purposive sampling approach was adopted to recruit participants. As for the inclusion criteria, they included (1) physicians consenting to participate and (2) physicians having left the country after October 2019 for the migrating doctors and doctors having decided to stay in the country for doctors staying in Lebanon. As for the exclusion criterion, it was related to the fact of having left the country before October 2019. The characteristics of doctors who have participated in the FGD are presented in Table 2.

**Table 2:** Characteristics of doctors participating in the FGD.

Key informant category	Sample
Physicians who emigrated or decided to emigrate	– Two (2) women and four (4) men. – The years of practice varied between seven years (7) and thirty-two years (32).
Physicians who decided to stay	– Three (3) women and three (3) men. – The years of practice varied between five years (5) and twenty-five years (25).

#### 2.2.3. Data analysis

The recorded FGD were transcribed, and notes from the unrecorded FGD were organized and then transferred to Dedoose for coding. Following this, EN and MM employed an inductive coding approach to identify recurring themes with the goal of categorizing these recommendations. After extensive coding and discussions, the research team collectively decided to classify the recommendations—aimed at maintaining the quality of care provision amidst the crisis—into four categories, encompassing the following:

1. Necessary Healthcare System Improvements.
2. Doctor Migration Understanding and Quantification.
3. Migration Mitigation and Slowdown Strategies.
4. Continuity of Care Contingency Plans.

### 2.3. Ethical considerations

All data collection methods adhered to ethical principles, ensuring privacy, confidentiality, informed and voluntary participation, and the best interests of the KI and participants. The researchers explicitly communicated to the KI and participants that they had the right to terminate the interview or withdraw from the focus group discussion FGD at any time without facing any consequences. Furthermore, the identities of the KI and participants were strictly protected throughout the entire data collection, analysis, and dissemination processes. The collected data is securely stored on the researchers' password-protected computers, with limited access only available to the researchers themselves. All recordings and transcripts will be deleted upon publication of the manuscript.

The study was reviewed and approved by the Saint Joseph University of Beirut Research Ethics Board on June 29, 2022 (reference number: CEHDF file 2037).

## 3. Results

### 3.1. Factors influencing physician's emigration

#### 3.1.1. Push factors

Numerous factors leading to emigration of doctors were identified through the semi-structured interviews conducted with migrant doctors, with the professional factors and political context playing a primary role in pushing physicians to emigrate. Furthermore, these aspects were found to be interconnected with the country's context, deeply affecting medical practice at various levels. In fact, the unstable country context coupled with a socio-economic crisis have led to devaluation in national currency which resulted in reduced doctors' remuneration and problems in importation of equipment and medical supplies which deeply affected the quality of care. With regard to the professional factors pushing doctors to emigrate, they were mainly related to reduced revenues, concerns about career paths, deteriorating quality of care, and the financial exploitation of patients, creating an unhealthy practice environment.

Firstly, physicians observed a significant decrease in their revenues compared to 2018. This decline was primarily due to unprecedented currency devaluation, delays in subsidies from third-party payers, reduced out-of-pocket expenses, a lower number of patients from outside Lebanon, and the majority of patients only seeking consultation in cases of emergency.

*“Firstly, financial reasons play a significant role because our current compensation is very low. As [specialty of the doctor], we receive payment in Lebanese Pounds, and due to the bank transfer process, we cannot even access the differences in the exchange rates. Consequently, my income is extremely low. Over the past two years, I have been earning barely five hundred dollars per month”. MD4*

Secondly, informants reported concerns related to their careers that pushed them to settle broad. These concerns were related to their career path, with various aspects mentioned by the informants: refusal to practice in a country with limited opportunities for professional development, limited experience due to the lack of continuing education opportunities, and the absence of specialized practice because of shortages in specialized equipment.

*"Imagine, at our hospital, they've stopped performing transplantations. Imagine that we're still using low-flux dialysis, something we abandoned three or four years ago, while in Dubai and Saudi Arabia, they're using a hyper-high-flux method. While we strive not to regress, even in this aspect, I have a feeling that we're stagnating. I want to challenge myself with something new”. MD9*

Thirdly, the reduced quality of care was also cited by physicians as a reason for their decision to leave. They explained that the deep impact of the socio-economic crisis on various aspects of the Lebanese health system, such as the availability of a skilled health workforce, necessary materials and equipment, and proper health system infrastructure, had weakened the health system and restricted the physicians. Regardless of a physician's skill, without a skilled multidisciplinary team and the necessary equipment and supplies, they would not be able to provide high-quality care to patients. Faced with their inability to fulfill their responsibilities towards patients, the physicians chose to leave.

*“There's a question of ensuring the equipment. We have responsibilities toward patients, and patients may not always understand that a doctor alone cannot handle and ensure everything”. MD9*

*“Basic supplies were sometimes unavailable, such as gloves, and we were experiencing shortages in certain qualities of sutures. The overall quality of our equipment and supplies was deteriorating, and we found ourselves heavily reliant on imports from other countries. Patients couldn't afford some of the necessary equipment for procedures like uteroscopy, including the uteroscope and laser fiber. Consequently, it became more challenging to perform these procedures in Lebanon”. MD13*

Finally, the financial exploitation of patients became evident during the crisis, exacerbated by the absence of proper governance in the health system. Each hospital sets its own prices and payment requirements for patients, leading to unacceptable exploitation of patients without any regulatory oversight. Some doctors witnessed this injustice and refused to be part of a system lacking rule of law. Consequently, they made the decision to leave.

*“The hospitals operate in a “black market” fashion, with each setting their own prices. I don't align with this approach. Recently, I was upset; for example, they asked me how much I wanted to charge if a patient is admitted. Why should I decide a price, whether it's a hundred, two hundred, or even three hundred? It should be standardized and codified”. MD16*

Professional factors were followed by factors related to the political environment. In fact, many participating doctors have stated that the Lebanese political system, leading to cyclical crises and in which change is difficult to achieve, is the reason for their departure. Furthermore, some of them expressed immense disappointment as neither the revolution nor the recent national elections had brought the slightest change. This political situation, combined with high levels of corruption, insecurity, and a lack of proper legal framework, drove many doctors to seek stability and settle abroad. Some informants mentioned that they managed to cope with the situation despite all the instability they experienced, until the Beirut port explosion on August 4th, 2020, which was the turning point that immediately made them decide to leave the country.

*“Starting from October 2019, the economic situation began to deteriorate. We thought we could continue like everyone else in Lebanon. However, the security situation became increasingly turbulent in the country, with protests and gunshot injuries seen at [name of hospital] and so on, the situation was not only unstable economically but also on the ground. Then came the explosion at the Port of Beirut. I was at [name of the hospital], and it was catastrophic. Even though my family was not directly affected physically, but psychologically especially after seeing the people around us, it was a catastrophe. What was even worse was the response, or lack thereof, from the so-called authorities in this country. So, this explosion was a turning point for us in terms of security”. MD12*

Also, concerning macro/contextual factors, the unparalleled socio-economic crisis affecting the country has not only impacted medical practice but has also affected doctors' abilities to meet their own and their families' basic needs. This was primarily the result of the national bankruptcy, which prevented doctors from accessing their savings, as well as fuel shortages, bread shortages, frequent power cuts, and unprecedented inflation, all of which have resulted in dreadful living conditions.

*“Everything was fine in Lebanon until about three years ago. I found that the situation had become unbearable in order to continue fulfilling the responsibilities required of a family man”. MD6*

*“The urgency arose because we were living in [name of the region], where it gets quite cold in winter, and it's really not viable if there's no electricity or heating fuel. So, at one point, we thought we had to spend the winter somewhere else. To share an anecdote, one day it had snowed in [name of the region], and there was no electricity, no heating, and it was really cold in the house with two children, one and three years old. That's not acceptable”. MD11*

Furthermore, the socio-economic crisis has jeopardized the education system as many schools and universities were unable to maintain their level of education given the huge migration of instructors and the reduced ability of caregivers to pay for their children's education. All these factors were cited by informants as reasons for seeking opportunities abroad that would allow them to maintain their incomes and be able to secure their families' basic needs.

*“So, at a certain point, you realize that yes, it's primarily a financial issue, but then, as my children reach four or five years old, there will also be academic security to consider. I'm not sure if the schools will be open”. MD8*

*“School fees have become a significant burden. I'm not sure if you're aware, but schools have started requiring payment in dollars. I believe that even minimal activity at the clinic or in the operating room could generate a reasonable income to lead an acceptable life in Lebanon, but my main concern is the school fees”. MD6*

In addition to reasons related to the professional, socio-economic, and political environment, doctors also mentioned factors related to their families as well as other personal considerations that influenced their decision. These factors included the desire for family and friends' reunions. Some doctors stated that they had to leave because their partners or children had decided to move to a safer country with better career opportunities. Others wanted to leave to reunite with their colleagues and university friends, many of whom had already left the country and created pathways and opportunities abroad.

*“I've already mentioned that the decision was primarily made by my husband rather than me”. MD14*

*“Furthermore, with the situation in Lebanon, my husband can no longer be present there all the time, so it's mainly for family reasons and family reunification”. MD1*

*“The first thing we did was ask our seven-year-old son if he was ready to live in [name of country]?” He said, “Yes”, because we went through difficult periods where my son had to see a psychologist. He became anxious; when the electricity cut off, he couldn't sit alone or take the elevator by himself. So, he was psychologically disturbed, and he's ready to go to [name of the country] because he knows things are not right in Lebanon”. MD7*

Other individuals' motivations for emigration were also cited and were interconnected with various factors. These included burnout due to the country's situation and the numerous crises faced, as well as the desire to move abroad to acquire foreign nationality or join international medical boards, offering a sense of security and stability.

*“The economic crisis, corruption in the country, corruption in the banks, corruption in the government—mentally, I am drained. It's not just about losing purchasing power; it's about all the corruption in the country. I want to leave, actually”. MD7*

*“At 9 p.m., I received a call from [name of country], and they told me that they would take me. I called [name of spouse] that night (the night of the Beirut port explosion) and asked if she was okay. She said yes, but she couldn't come because the roads were blocked. So, I told her that the offer from [name of country] was to leave. She said, “Look, you're signing the contract without even reading it”, and I agreed with her because today it was a message that we needed to leave, despite how difficult it is to leave with my parents being ill in Lebanon”. MD3*

*“The primary motivation behind my decision is to attain a certain professional freedom, to acquire a [name of the country] board, in order to have a sense of security. I need to have a solid Plan B, and that's what motivates my move to [name of the country] the most—having a strong backup plan, a country where I can live, of course, that's the primary goal”. MD5*

Another cross-cutting theme between the macro and micro levels emerged and was related to the broken Lebanese heath system. Many doctors revealed that the lack of treatments and medications led them to consider leaving the country to secure better health care for their family members. In fact, one doctor stated that shortages in vaccines made it very difficult for him/her to provide proper and timely immunization for his/her children. Another doctor stated having a chronic condition requiring close monitoring and treatments that are not available anymore in Lebanon which pushed him/her to settle abroad in a country where his/her medical condition could be well-managed.

*“It was primarily for the safety of our children, be it food security, medical security, and we couldn't find vaccines easily. We had to adjust the vaccination schedule. So, at one point, we thought that for the children, it's better to leave, even if it puts my career at risk”. MD11*

Finally, when asked about their intentions to return to Lebanon, two opinions prevailed: a few doctors considered that returning to Lebanon is so difficult and even impossible, whereas others expressed their wish to return if the country situation improves or their personal circumstances change. At the national level, they wanted an improvement in the socio-economic situation, a better political system with greater accountability, less corruption, but above all less uncertainty for the future. As for their personal situation, the doctors considered that having a second nationality or a work permit in a foreign country facilitating their quick professional shift would help them feel more secure in case of a return to Lebanon.

*“A stable country like it was before, knowing what the Lebanese pound will be worth, knowing that people won't be afraid to come for surgery in Lebanon anymore, knowing that my other friends will also return because we need to feel…I can't live alone; I don't have anyone left in Lebanon. Lebanon needs to resemble what it was before 2018, I believe, for me to take a new risk and start a new career”. MD9*

#### 3.1.2. Stay and stick factors

Interviews conducted with doctors who decided to stay in Lebanon helped identify the reasons for their decision, and which were mainly meso- and micro-level factors and related to emotional attachment and belonging to both their professional environment and the country.

At the professional level in Lebanon, doctors who have opted to stay have experienced certain improvements in their working conditions, although these conditions remain less than ideal. While these improvements have allowed them to sustain a decent standard of living, they are far from optimal. These positive changes include an increase in physicians' income, enabling them to meet their families' basic needs. However, this amelioration in working conditions has led to a two-tiered healthcare system, where high-quality care is accessible to those who can afford it, creating what can be described as a “cash economy” solution. The socio-economic crisis has also led to significant disparities among different medical specialties, with certain fields such as ophthalmology being severely affected due to medication shortages. Consequently, some doctors in these specialties face challenges in their practice, while others remain relatively unaffected. Furthermore, these disparities extend beyond specialties to regional differences, with notable variations in the quality of healthcare services between Beirut, the capital, and peripheral regions. This regional imbalance exacerbates existing inequities in the healthcare system. Despite these challenges, the physicians who have chosen to remain in Lebanon stated having managed to adapt to the circumstances, but their ability to provide quality care is hindered by these constraints.

*“The salary situation is getting better; we're still far from our pre-crisis situation, but we're no longer at the bottom of the crisis; we're starting to emerge, at least in terms of the hospital and the university”. SD7*

*“If we continue to be compensated in this way, it's fine; we manage. I'm not going to make a fortune; anyway, I didn't choose medicine for that. If I can still manage and meet my own needs and the needs of my family, because that's important”. SD5*

Another significant professional factor keeping doctors in Lebanon is their satisfaction and motivation at work, often stemming from strong relationships with patients or a positive and supportive work environment. For instance, some informants mentioned having experienced appreciation and value in their workplace, and their superiors have improved their conditions and offered incentives to dissuade them from considering opportunities abroad. Additionally, many doctors reported having invested years in successful careers, making it difficult for them to sacrifice their hard-earned positions for a new start elsewhere. Furthermore, certain doctors feel a sense of responsibility for their hospitals, teams, and patients, making leaving a difficult choice.

*“I am part of a team where I am content, I feel secure, and I get along well with other team members, whether it's at the university or the hospital. I also don't have any personal conflicts that would push me to leave. So, all of these factors need to be considered in someone's decision to leave a position and go elsewhere”. SD8*

*“I have a lot of patients, and my career has been progressing well. I didn't want to leave it all behind and quit”. SD3*

*“I admit that if I weren't responsible for these interns, for these residents, I would have been more inclined to do it (emigrate)”. SD1*

*In terms of the broader health system, staying doctors believe that the worst of the crisis is over, and they perceive gradual improvements, which influence their decision to stay. This combination of professional fulfillment, loyalty, and a cautious optimism about the future of the healthcare system keeps these doctors in Lebanon, despite the persisting challenges. “Now, as work resumes, working conditions are improving, whether at the university or the hospital, the idea (of emigrating) no longer crosses my mind, as long as conditions continue to improve”. SD5*

At the micro level, various personal and family factors influence doctors' decisions to stay in Lebanon. Family commitments, including partners and children, often play a crucial role; some doctors stay due to their partner's decision or to ensure their children's social stability. Personal attachments and a sense of belonging to Lebanon also factor in, with some doctors citing their love for the country as a reason to endure challenging living conditions. Additionally, some doctors remaining in Lebanon perceive their decision as an act of resistance, showcasing their determination to navigate the challenges and contribute to their country's well-being despite the adversities.

*“And then there are our parents, whether we like it or not, leaving them all alone in such a crisis is not something that's easy. I mean, our parents still need us during such a crisis”. SD2*

*“Because I think we need to resist somewhere. And I believe we need to set a good example for these young people who have only one desire, which is to leave”. SD9*

Many of the remaining doctors said they had considered leaving at one point but decided to stay after assessing the pros and cons of emigration. Nevertheless, most doctors agreed on the factors that will make them reconsider their decision to stay in the country: aggravation of the socio-economic crisis and the onset of war. In fact, informants reported an improvement in the socio-economic situation that is likely to continue. Nevertheless, they assured that if the crisis escalates leading to difficulties in providing basic needs such as food, electricity, education, etc. they will definitely leave the country. Moreover, when asked about the reason that would make them immediately leave, the only answer received was “onset of war and armed conflict”, considering that they could handle anything but war.

*“One, armed conflicts because I've long decided not to shed a drop of blood for this country. Two, the education system. And three, if despite my work, I can't provide the basic necessities for my family, for my family's life”. SD7*

### 3.2. Key findings and recommendations from the FGD

During the FGDs, participants actively contributed to formulating recommendations, drawing from the findings of the semi-structured interviews. These recommendations were then categorized by the researchers into four distinct groups.

#### 3.2.1. Necessary healthcare system improvements

Participants stated that physicians represent a vital component of human resources for health, one of the interconnected pillars constituting a comprehensive health system. It is therefore particularly important to tackle the migration problem using a holistic approach. For instance, the migration of doctors is the result of the various crises facing the country and leading to a fragile system, not providing doctors with the necessary resources—such as adequate infrastructure, necessary human resources (i.e., nurses), equipment and medications, etc.—to provide quality care services. Therefore, participants considered that any future strategy for the health system should be a participatory strategy bringing together all stakeholders to address the problem. Participants emphasized that the panoply of stakeholders allows a better understanding of the different causes of the problems as well as the identification of the bottlenecks for improvement. Mentioned stakeholders were the Ministry of Public Health, the Parliamentary Health Committee, health care service providers, health financiers (including representatives of public funds, representatives of private insurance, funders and donors), producers of health goods and services (including the pharmaceutical industry, and importers of medicines or medical devices) and service users. This bottom-up, participatory decision-making approach is meant to provide realistic, achievable, and relevant solutions leading to system improvements that will result in slower physician migration.

*“The effectiveness of a doctor, regardless of their skill level, depends on a well-functioning system equipped with necessary tools and supported by a team comprising nurses and other specialists”. FGD 1- Participant 3*

#### 3.2.2. Doctor migration understanding and quantification

Participants emphasized that physician migration unequally affects different regions of the country and various medical practices and specialties. For example, urban areas are witnessing a higher rate of physician migration compared to rural regions because physicians in urban settings are generally more qualified and, therefore, more likely to find appealing job opportunities abroad. However, participants pointed out that urban regions already have a surplus of physicians compared to pre-crisis needs. Therefore, there is a need for accurate quantitative mapping of physician distribution and the impact of migration to gain a better understanding of the situation and identify areas at high risk of shortages.

Furthermore, physician migration affects different specialties in varying ways; some specialties are already experiencing severe shortages, while others are less affected. This discrepancy can be attributed to the initial high number of doctors in certain specialties.

To comprehensively grasp the issue of migration and its impact on healthcare provision, participants recommended establishing a national committee. The committee's primary mission would be to map available resources, including the number of doctors, their specialties, and their distribution nationwide and between the private and public sectors. This approach would enable the identification of specialties at high risk of human resource shortages and the development of targeted strategies to enhance physician retention. In fact, one participant considered that the current healthcare system continues to prioritize specialists, even in specialties and regions where there is already an excess of doctors. Consequently, this participant believes that the impact of this migration will be less significant in specialties with a surplus, unlike in areas where there were only one or two specialists available in the country.

“*The impact of this migration varies across different specialties and regions. It's crucial to comprehend these variations to develop effective mitigation strategies for specialist shortages. Additionally, it's important to note that in several specialties and regions, we already had an excess of physicians”. FGD 1- Participant 4*

#### 3.2.3. Migration mitigation and slowdown strategies

When asked about strategies to retain physicians in the system and slow the pace of migration, participants mentioned different strategies that varied in scope and nature. In terms of scope, the first recommendation at the national level was directed to the Ministry of Public Health and was to endorse the code of practice to prevent further and unethical exhaustion of available resources. In addition, participants again emphasized the importance of health system reforms and improvements as a trigger to retain doctors or bring them back into the country. In fact, these improvements contribute directly to improving the working conditions and working environment of doctors, which are important causes of migration. In addition to national-level interventions, participants reiterated the importance of targeted strategies to increase physician retention that focus on specialties at high risk of human resource shortages.

“*The Ministry of Health has a significant role to play, particularly in endorsing the code of ethics to prevent a complete drain in certain specialties”. FGD 1- Participant 2*

As for the types of interventions, they could be divided between financial incentives and in-kind incentives. In terms of financial incentives, participants called on (1) medical institutions to provide support plans for doctors, such as direct financial support, tuition and university fees for children, medical insurance, etc. (2) health funders to revise the scale of compensation in light of national currency devaluation and reduce the delays in reimbursement of fees, and (3) funders (such as United Nations agencies and non-governmental health support organizations) to consider the development of programs and initiatives aimed at providing financial support directly to health care providers rather than channeling their support through institutions and users of services. In terms of in-kind incentives, these mainly include national and institutional recognition and enhancement. For example, at the national level, the media for the past year has focused only on “medical malpractice”, creating a relatively hostile public opinion towards doctors, and making them feel unrecognized. Therefore, the media was invited to highlight the doctors who, despite inadequate professional conditions, have remained; this recognition is believed to affect their decision to stay and not to migrate.

“*We often feel offended, especially in these times of crisis, when people cannot afford necessary medical treatments. Furthermore, the national media contributes to this offense by magnifying minor incidents involving doctors instead of acknowledging our efforts. It's crucial to understand that feeling acknowledged is just as vital as financial gains for many of us”. FGD 1- Participant 1*

#### 3.2.4. Continuity of care contingency plans

Physicians participating in focus group discussions emphasized that suggestions on improving the health care system, developing and implementing human resource retention strategies could not be achieved overnight and may not have drastic short-term impact. Therefore, prompt actions and alternatives are needed to ensure continuity of quality care, especially in specialties where disruption of care due to specialist shortages has already occurred. These alternatives include the organization of short-term medical missions involving emigrant doctors, the implementation of telemedicine, special recruitment strategies, targeted training, and task-shifting approaches.

Participants highlighted that short-term missions are commonly used to compensate for doctor shortages at national and sub-national levels in many countries. This strategy is intended to be implemented only in specialties where disruption of care has already occurred, but mainly those requiring surgical interventions such as cardio-pediatric surgery. This approach is seen as an effective mitigation strategy, particularly for elective cases not requiring immediate intervention. However, participating physicians stated that certain elements must be taken into consideration to ensure success, such as considering securing a minimum financial package for deployed doctors, such as travel, transport, accommodation, and doctor's fees if necessary. Another element has been raised by doctors practicing abroad and is that of inviting Lebanese institutions, the Lebanese ministry of public health and the Lebanese order of physicians to initiate negotiations and develop agreements with institutions abroad to prevent these missions from replacing all their holidays and having them considered as regular paid working days.

“*I am willing to undertake short-term missions in Lebanon to help my fellow citizens, even if the pay is not as high as in other countries. However, a challenge arises as I would have to utilize my off days, leaving me with no remaining time off. Hence, agreements between stakeholders in Lebanon and my current workplace, allowing me to travel without it being deducted from my leave, would be a significant opportunity for me”. FGD 1- Participant 5*

Telemedicine, which is becoming increasingly popular in many fields of medicine, was considered a very effective alternative to counter the shortage of doctors. Telemedicine can, for example, be implemented in many fields that do not require physical consultation, which lacks specialists, such as neuroradiology, anatomo-pathology, medical dermatology, etc. These consultations can be provided both by doctors practicing in Lebanon or residing abroad which allows securing sufficient human resources. For instance, doctors who have migrated have shown their willingness to devote time to online services for this will help to benefit their country and its community.

Participants also noted that recruitment strategies focused on freshly graduated physicians could help secure sufficient resources to meet the need. For instance, one participant in the second FGD highlighted that freshly graduated doctors face difficulties in finding a suitable position in any Lebanese medical institution. Therefore, he emphasized the importance of retaining them within the system and training them to fill the existing gaps especially that they are more likely to accept lower salaries if they are offered satisfactory positions.

In terms of training, a mapping of the medical human resources available (mainly doctors) makes it possible to identify the specialties at high risk of shortage of doctors or which have already been confronted with shortages of doctors. This resource mapping will help inform national universities with medical schools about the needs of the “market” to orient their medical students towards particular specialties. Furthermore, this situation analysis in terms of available resources will help advocate with national universities to organize medical education in a suitable way to mitigate this downward trend in the number of medical students. For example, universities can reduce the number of students accepted for certain specialties with an excess of human resources. Finally, the approach of task shifting to less specialized staff adopted in many contexts with shortages of specialized human resources was also mentioned by participants as a potential solution. This approach can be achieved by the transfer of tasks from physicians to other health professionals (such as nurses) in primary health care settings and from sub-specialists to specialists at the tertiary care level. However, this approach requires the organization of training, and close monitoring to ensure continuity but also the quality of care.

## 4. Discussion

The findings of the study illustrate that the decision to migrate is influenced by multiple interrelated factors. Among these factors, meso-level factors or professional considerations emerge as the primary drivers pushing doctors to explore employment opportunities abroad. These include declining income, career problems, reduced quality of care and unhealthy work environment. Professional factors were followed by macro-level national factors such as deteriorated political and socio-economic contexts leading to instability and insecurity pushing doctors to leave the country. Micro-level or personal factors related to family commitments and the necessity to provide basic needs for family members had also led some physicians to decide to migrate. The drivers of migration identified in this study largely align with those described in the literature (2, 5, 6, 9, 20). These drivers primarily revolve around challenges related to meeting material expectations and basic needs, job insecurity, worries regarding personal and family safety, aspirations for an improved professional future, and the belief in superior opportunities overseas. Therefore, this migration was somehow expected yet surprisingly harsh. In fact, the current migration wave was a particularly dangerous one given its large scale, rapid upward trend as well as the types of resources it affects. In fact, the Lebanese crisis has led to an imbalance, with the pushing factors having taken over the ones keeping doctors in the country. This unprecedented migration coupled with an already fragile health system has posed a high risk of disrupting the supply of care. Moreover, the current migration seen by participants as draining the most qualified and young doctors is likely to result in a catastrophic decrease in the expertise available if not promptly addressed by retention strategies. However, it is noteworthy that some specificities have emerged. Notably, factors related to post-graduate training, such as unfair competition for training positions, and unclear curriculum, were absent in our findings. This stands in contrast to the existing literature, notably the study conducted by Al-Khalisi, which underscored training-related concerns as a significant driver behind the migration of physicians in Iraq (21). This suggests a quality of training at the country level despite the crisis or a lack of interest among physicians in these particular aspects of medicine. Furthermore, despite the numerous challenges faced by the country's health system, the fear of contracting a serious illness in Lebanon was primarily linked to shortages in mandatory childhood vaccines rather than the COVID-19 pandemic. This finding is particularly significant, given the timing of the study during the pandemic. It indicates that the shortage of essential vaccines for children played a more influential role in motivating migration than the immediate threat of COVID-19. However, it’s possible that the same risks were perceived to exist abroad, given the global scale of the pandemic. Additionally, despite Lebanon's status as a parliamentary democratic republic, it is a “sectarian power-sharing” system, with underlying sectarian tensions, yet “ethnic discrimination”, and “religious conflicts”, did not emerge as significant drivers pushing physicians to leave the country.

With regards to the factors that retain physicians in the country, they are primarily associated with affective attachment and a sense of belonging to their professional environment and the nation. Meso-level factors including the recognition and appreciation of doctors, both by institutions and patients, have served as crucial factors in retaining them. Furthermore, many doctors expressed that they had made significant progress in their careers and were not willing to abandon everything to work abroad. As for the micro-level factors, family commitments, such as a partner's decision to stay and the well-being of children, have also played a role in retaining a few doctors within the country. Therefore, evidence reveals that despite facing non-ideal working conditions far from pre-crisis standards, some doctors have opted to stay in Lebanon. Their persistence stems mainly from a modest improvement in working conditions, enabling them to maintain a decent standard of living, coupled with their belief in a gradual and continuous improvement. This contrasts with the sentiments expressed by some emigrant doctors, who lack confidence in the national and healthcare systems. Other factors such as positive patient relationships, supportive work environments, and established successful careers were identified in the study as influential for retaining doctors in Lebanon but played no significant role for those who decided to emigrate. In fact, doctors staying in Lebanon exhibit greater persistence and attachment to their profession and country, diverging from the rational decisions of emigrant counterparts who present strong evidence of the system's deterioration compelling them to leave. The interview findings and reflections from focus group discussions underscore the interconnections among the drivers of physician migration. Moreover, the crisis in Lebanon was observed to impact all levels as described by Hajian et al. (9), with the deterioration of macro-level factors significantly influencing meso-level factors, which in turn exerted a substantial impact on micro-level factors. As for the recommendations, aimed at reducing the migration flow and mitigating the crisis's impact, they primarily emphasized addressing the entire healthcare system to the greatest feasible extent. Physicians' needs extend beyond financial incentives or specific privileges; they require a functioning system in which they can professionally advance and find satisfaction in both the quality and manner of healthcare service delivery. The main recommendations suggested to address the migration flow encompass implementing national health system reforms, adopting evidence-based strategies to retain human resources, negotiating with recruiting institutions to endorse the code ethics ending unethical practices draining countries' human resources, providing financial incentives, and valuing and recognizing physicians. Rapid interventions are also needed to prevent increased morbidity and mortality that could result from physicians' shortages. They comprise short-term medical missions involving specialists from specialties facing catastrophic shortages, telemedicine as an alternative for services that can be provided online, and the adoption of the task-shifting approach to reduce the workload on overburdened specialists.

The findings of this study—which highlighted the different factors influencing migration while framing them in the Lebanese context—should help inform key stakeholders and policy makers about the needed interventions to restore the quality of care. The recommendations formulated through the FGD, even if they are deemed highly relevant, their feasibility strongly depends on a multitude of factors, with political willingness being the most crucial one. Collaborative and participatory governance of the health system is essential to enable bringing together all stakeholders, including physicians, to better understand the bottlenecks that prevent them from staying in the system and to solve these problems through evidence-based bottom-up decision-making and the mobilization of necessary resources.

### 4.1. Strengths and limitations

This study has several strengths and some potential limitations. With regards to strengths, the qualitative research methods employed as well as the categories of participants selected enabled the researchers to explore physician's migration from different perspectives allowing a comprehensive understanding of the different factors influencing migration as well as their interconnections. Moreover, the context in which the study was carried out is exceptional, with Lebanon being a country having simultaneously experienced an unprecedented socio-economic crisis, bankruptcy, political instability, security problems and the third largest non-nuclear explosion, killing hundreds and injuring thousands. As for the limitations of the study, the position and the identity of the investigator (familiar to most participants) might have influenced their statements leading to prevarication and information biases. Nevertheless, several approaches were adopted to mitigate this bias, including adhering to ethical considerations, providing participants with the right to withdraw from the study at any point during the data collection process, having two researchers collaborate on coding and analyzing the findings, and employing a standardized approach guided by robust frameworks retrieved from the literature. Furthermore, a selection bias was identified with most participants practicing in urban rather than rural settings resulting in an underrepresentation of physicians working in rural settings and a reduced generalizability of the results. Additionally, even though the research did not uncover any evidence of gender discrimination playing a role in physicians' migration, there was an absence of in-depth examination regarding how the gender of the participants might have shaped their decision-making factors. Finally, despite deriving interesting recommendations, the researchers believe that more in-depth information could have been collected and the implementation of these recommendations discussed if additional FGDs had been organized or if co-validation workshops had been conducted with key stakeholders, including the Ministry of Public Health, the Order of Physicians, etc.

## 5. Conclusion

The emigration of doctors and its drastic impact on the supply of care and the health outcomes of the Lebanese community is becoming an issue of growing concern. This study explored the factors leading to this emigration, the factors retaining doctors in the country as well as suggested solutions to limit physician migration and mitigate its consequences. Although the proposed solutions are evidence-based and geared towards addressing the issues leading to and resulting from this migration, actual improvement seems to depend heavily on the political will to create some stability in the country and the governance model of the health system that could lead to sustainable solutions and provide guarantees that increase doctors' trust in the system.

## Data Availability

The datasets presented in this article are not readily available because the data is only available to the researchers themselves. Further inquiries can be directed to the corresponding author.
