# Supplementary material for: Exodus of Lebanese doctors in times of crisis: a qualitative study
Source: Front Health Serv. 2023 Oct 30;3:1240052. doi: 10.3389/frhs.2023.1240052 (PMC10643131; doi:10.3389/frhs.2023.1240052)
Supplement: Supplementary file 1 [file Datasheet1.pdf]

Topic guide: Doctors abroad / who decided to leave

Thank you for agreeing to participate in this study aiming to understand the factors that pushed some physicians to migrate or consider migrating, as well as the factors that make other physicians stay in Lebanon.

I'd like to start by introducing myself: ……….

THEMES	QUESTIONS
PARTICIPANT PROFILE	**Could you please introduce yourself, your educational background, your current position, the number of years of experience you have?**<br><br>*Do you have a 2nd citizenship or permanent residency?*
FACTORS THAT LEAD SOME PHYSICIANS TO MIGRATE OR CONSIDER MIGRATION	**What are the main factors that affected your decision?**<br><br>**Macro-level or national factors**<br>1. To what extent did the economic situation in Lebanon affect your decision to leave the country?<br>2. To what extent did the Lebanese political crisis affect your decision?<br>3. What factors or characteristics of the Lebanese health sector pushed you to decide to work abroad, if any? What factors or characteristics of the health sector in the country you are currently working in pushed you to choose that particular country, if any?<br>4. Did any of the social factors that I will cite affect your decision and in case it did, how did it affect your decision?<br>*Gender inequalities, ethnic and racial discriminations, low quality of social services, culture of migration and social pressure, poor social rights, and religious conflicts.*<br><br>**Meso-level / Professional factors**<br>1. Did the work conditions affect your decision to leave the country?<br>2. Was your decision affected by the educational environment in Lebanon (if applicable)?<br><br>**Micro-level / Personal factors**<br>1. Was your decision influenced by a desire/need for personal fulfillment?<br>2. Was your decision affected by your family conditions or any family concerns?
IDENTIFY MEASURES AIMING TO PRESERVE AN	**What are the factors that would make you or those who migrate consider returning to Lebanon?**

ADEQUATE PROVISION OF CARE.	**What are your recommendations for a continuum of quality care service provision in Lebanon in the light of the current crisis?**
	**Would you like to add anything?**

Thank you for your time

Topic guide: Doctors who decided to stay in Lebanon

Thank you for agreeing to participate in this study aiming to understand the factors that pushed some physicians to migrate or consider migrating, as well as the factors that make other physicians stay in Lebanon.

I'd like to start by introducing myself: ……….

<table>
<tr><td>THEMES</td><td>QUESTIONS</td></tr>
<tr><td>PARTICIPANT PROFILE</td><td>Could you please introduce yourself, your educational background, your current position, the number of years of experience you have?

Do you have a 2nd citizenship or permanent residency?</td></tr>
<tr><td>FACTORS THAT LEAD SOME DOCTORS TO STAY</td><td>What are the main factors that affected your decision?

Macro-level or national factors
1. Why didn't you think about migration despite the multiple crises Lebanon is facing?

2. What factors or characteristics of the Lebanese health sector pushed you to decide to stay, if any? What health sectors' factors or characteristics in other countries pushed you to choose to stay in Lebanon, if any?

3. Did any of the social factors that I will cite affect your decision and in case it did, how did it affect your decision?
Gender inequalities, ethnic and racial discriminations, low quality of social services, culture of migration and social pressure, poor social rights, and religious conflicts.

Meso-level / Professional factors
1. Was your decision affected by the educational environment in Lebanon (if applicable)?

2. Did the work conditions affect your decision to stay in the country?

Micro-level / Personal factors
1. Was your decision influenced by a desire/need for personal fulfillment?

2. Was your decision affected by your family conditions or any family concerns?</td></tr>
</table>

IDENTIFY MEASURES AIMING TO PRESERVE AN ADEQUATE PROVISION OF CARE.	**In your opinion, what are the factors that would make those who migrate consider returning to Lebanon?**<br><br>**What are your recommendations for a continuum of quality care service provision in Lebanon in the light of the current socio-economic crisis?**
	**Would you like to add anything?**

Thank you for your time

Topic guide for the Focus Group

Welcome and thank you for agreeing to participate in this study.

Start by explaining the context:

As part of my thesis in "Master in Health Management", I opted to study the exodus of Lebanese doctors with 3 objectives

- Identify the factors that push some physicians to migrate or consider migrating.
- Identify the factors that lead some physicians to stay.
- Identify measures to maintain an adequate supply of care.

Sharing results:
Twenty-eight interviews were carried out, involving 19 physicians who had emigrated and 9 physicians who chose to remain in Lebanon. The interview findings unveiled the following:

[EN presented the interview findings following the same structure as the results presented in the manuscript, discussing the factors influencing physicians' emigration, starting with the “Push factors” and then addressing the “Stay and Stick factors”.]

Then open question:
Today, we will focus together on identifying measures to maintain an adequate supply of care.
So, based on those results, what, in your opinion, can be done to maintain this supply of care?

If concordance, work out together.

Otherwise, reinject suggestions.

Tips:
- Never interrupt the participants.
- Do not insist.
- Do not suggest.
